# The effect of age and sex on T1, T2, and T2* relaxation time constants in cardiac MRI in healthy Finnish population

**DOI:** 10.1177/20584601261418629

**Published:** 2026-01-21

**Authors:** Mimmi K. Liukkonen, Miska Jämsä, Suvi Hartikainen, Minna Husso, Marja Hedman, Heikki Hietanen, Martin Ugander, Saara Sillanmäki, Elias Ylä-Herttuala

**Affiliations:** 1 Clinical Imaging Centre, 60650 Kuopio University Hospital, Kuopio, Finland; 2 School of Medicine, Faculty of Health Sciences, University of Eastern Finland, Kuopio, Finland; 3 Diagnostic Center, Helsinki University Hospital, Helsinki, Finland; 4 Institute of Clinical Medicine, University of Eastern Finland, Kuopio, Finland; 5 Department of Clinical Physiology, 27106 Karolinska University Hospital and Karolinska Institutet, Stockholm, Sweden; 6 Kolling Institute, Royal North Shore Hospital and University of Sydney, Sydney, New South Wales, Australia; 7 A.I. Virtanen Institute, University of Eastern Finland, Kuopio, Finland

**Keywords:** Magnetic resonance imaging, tissue characterisation, T1, T2, T2*, sex-related differences

## Abstract

**Background:**

Magnetic resonance imaging (MRI) enables the non-invasive assessment of myocardial tissue properties through the T1, T2, and T2* relaxation mapping. Establishing population-specific normal reference values enhances diagnostic accuracy.

**Purpose:**

To study the effect of sex and age on the T1, T2, and T2* relaxation time constants in a healthy Finnish population.

**Methods:**

We recruited 47 healthy volunteers aged 18–60 years from Eastern Finland from 2023 to 2024 and categorised them by sex and age (18–30 years, 31–41 years, and 42–60 years). The participants underwent a comprehensive screening process to eliminate the possibility of cardiac disease. MRI scans were conducted on 40 participants at 1.5 T. The T1, T2, and T2* relaxation time constants were calculated for basal, mid-ventricular, and apical short-axis slices.

**Results:**

The T1 and T2 relaxation time constants were higher in females than males (T1: 1040 ± 29 vs 1020 ± 17 ms, *p* < .01; T2: 51 ± 4 vs 48 ± 3 ms, *p* < .001). The 95% normal T1 range was 981–1098 ms for females and 985–1054 ms for males. The normal T2 range was 44–58 ms for females and 43–53 ms for males. No sex differences were found in the T2* relaxation times. The septal T2* across the whole population was 36 ± 7 ms (95% normal limit: 22–49 ms).

**Conclusion:**

This study established age-independent and sex-specific reference values for the native myocardial T1, T2, and T2* relaxation time constants at 1.5 T. Females had higher T1 and T2 values than males, and age did not affect these values.

## Introduction

Cardiovascular diseases (CVDs) are the leading cause of global mortality.^1^ Various types of CVDs lead to pathological alterations in the heart muscle. These conditions might cause myocardial oedema, inflammation, fibrosis or, in some cases, iron accumulation in the myocardium.^2–4^ Early detection of these pathological changes allows for more effective patient management.^5–7^ Non-invasive magnetic resonance imaging (MRI) provides a reliable assessment tool to characterise these myocardial tissue properties and the structural details of the myocardium.^8–10^

Cardiovascular MRI (CMR) is considered the reference standard for a non-invasive assessment of cardiac morphology and systolic function.^11,12^ In addition, the T1, T2, and T2* mapping techniques are myocardial tissue characterisation techniques that determine the various pathologies behind different CVDs.^13^ For example, the T1 relaxation time is based on the relaxation of the longitudinal component of magnetisation, and its mapping technique is used to characterise and quantify myocardial fibrosis by noting the increase in the T1 relaxation time constant.^13,14^

Diffuse myocardial fibrosis is associated with several cardiac conditions, including hypertrophic and dilated cardiomyopathy, and it is also closely linked with the progression of heart failure.^15–18^ T1 maps can also be used to determine focal fibrosis and scarring, such as resulting from myocardial infarction.^19^ Prior studies have demonstrated that native T1 and extracellular volume are influenced not only by underlying disease, but also by sex and age, including interactions between these factors, as well as by cardiac chamber size and heart rate.^20^ T2 relaxation time reflects the decay of the transverse component of magnetisation, and T2 mapping is used to for example assess myocardial oedema by detecting an increase in the T2 relaxation time constant.^21,22^ Myocardial oedema can be a sign of pathologies such as acute ischaemia and inflammatory diseases such as sarcoidosis and myocarditis.^23,24^ T2* relaxation also considers both the transversal decay of magnetisation and magnetic field inhomogeneities.^10^ T2* mapping is typically used to determine myocardial iron overload,^25,26^ for example, in patients receiving multiple blood transfusions or in conditions such as haemochromatosis.^10,27,28^ Iron deposition with low T2* values can also occur focally in intramyocardial haemorrhages due to acute myocardial infarction.^29^

Several studies have established normal values for the T1, T2, and T2* relaxation time constants in healthy myocardium.^30–34^ Despite previous studies, there is a need for device-, protocol-, and population-specific normal values to ensure maximal diagnostic accuracy.^35^ Establishing the physiological T1, T2, and T2* relaxation time constant values is critical to differentiate healthy and abnormal cardiac tissue in clinical practice.

Our primary aim was to study the effect of sex and age on CMR tissue characterisation values, and simultaneously to establish reference values for the T1, T2, and T2* relaxation time constants using a 1.5 T system (Siemens Magnetom Sola) for a Finnish population. We hypothesised that the myocardial T1, T2, and T2* relaxation time constants would exhibit significant variations between sexes and across different age groups. These variations could influence clinicians’ interpretations of imaging data.

## Methods

### Study population

This study was conducted in accordance with the ethical standards set forth by the Declaration of Helsinki and was approved by the ethics committee of Wellbeing Services County of North Savo (approval number 21/2023). Informed consent was obtained from all participants.

We recruited 47 healthy Caucasian volunteers from Eastern Finland from 2023 to 2024. The inclusion criteria were age between 18 and 60 years old. Exclusion criteria included any known cardiovascular disease, known hypertension, diabetes mellitus, dyslipidemia requiring pharmacologic treatment, renal impairment, or systemic inflammatory disorders. Participants were thoroughly screened to ensure their eligibility. This included a resting electrocardiogram (ECG) and blood tests (including troponin, creatine kinase-MB, B-type natriuretic peptide, haemoglobin, low-density lipoprotein, and glycated haemoglobin (HbA1c) to rule out electrophysiological abnormalities and overt cardiovascular risk factors. A medical specialist assessed the clinical status, including cardiac auscultation and blood pressure measurements. An echocardiogram was then performed to rule out functional or structural abnormalities (study subjects had to have a normal cardiac anatomy, normal systolic and diastolic function; no significant valvular regurgitation or stenosis; and normal myocardial wall thickness and regional wall motion). Seven participants were excluded due to abnormalities in the prescanning phase. CMR was conducted on 40 subjects with normal ECG, echocardiography, and laboratory tests.

### MRI

MRI scans were performed using the 1.5 T Siemens Magnetom Sola (version XA 31, Siemens Healthineers, Forchheim, Germany) MRI device. The T1, T2, and T2* relaxation time maps were gathered with ECG triggering and breath holding. Relaxation time maps were performed as three short-axis images from the basal, mid, and apical sections. T1 mapping was imaged using optimised Modified Look-Locker Inversion Recovery (MOLLI), a 5(3)3 prototype (5 acquisition heartbeats followed by 3 recovery heartbeats and a further 3 acquisition heartbeats) sequence, while balanced Steady-State Free Precession was used as the T2 mapping sequence, and a multi-echo gradient echo sequence was used as the T2* mapping sequence. Matrix size was selected based on the distance between two R-peaks (R-R interval) of the participants (144 × 256 mm^2^ for R-R >700 ms and 380 × 420 mm^2^ for R-R <700 ms). The parameters of the T1, T2, and T2* mapping sequences are presented in Table 1. The T1, T2, and T2* maps were generated in line on the Siemens scanner, and all quantitative measurements were performed using Circle Cvi42 v. 6.0 (Circle Cardiovascular Imaging Inc., Canada). No participants were completely excluded after CMR due to low-quality maps. The first five participants had missing T2* values for all regions because we did not include T2* at the beginning of the study in the imaging protocol. In one additional case, T2* values could not be measured at the basal level of the heart, although values from other myocardial regions were available. Gadolinium enhancement was not included in the CMR protocol.

**Table 1.** Parameters for T1, T2, and T2* mapping sequences.

T1 Mapping sequence parameters	TR (Repetition time)	299 ms
	TE (Echo time)	1.08 ms
	Flip angle	35°
	TI	186 ms
	Slice thickness	8 mm
	FOV (Field of view)	402 × 402 mm
	Matrix	256 × 169
	Bandwidth	1085 Hz/Px
T2 Mapping sequence parameters	TR	203 ms
	TE	1.18 ms
	Flip angle	70°
	Slice thickness	5 mm
	FOV	400 × 329 mm
	Matrix	192 × 146
	Bandwidth	1184 Hz/Px
T2* Mapping sequence parameters	TR	700 ms
	TE (1-8)	2.08, 4.10, 6.12, 8.14, 10.16, 12.18, 14.20, 16.22 ms
	Flip angle	20°
	Slice thickness	8 mm
	FOV	400 × 325 mm
	Matrix	256 × 153
	Bandwidth	814 Hz/Px

### Data analysis

**Fig 1.**
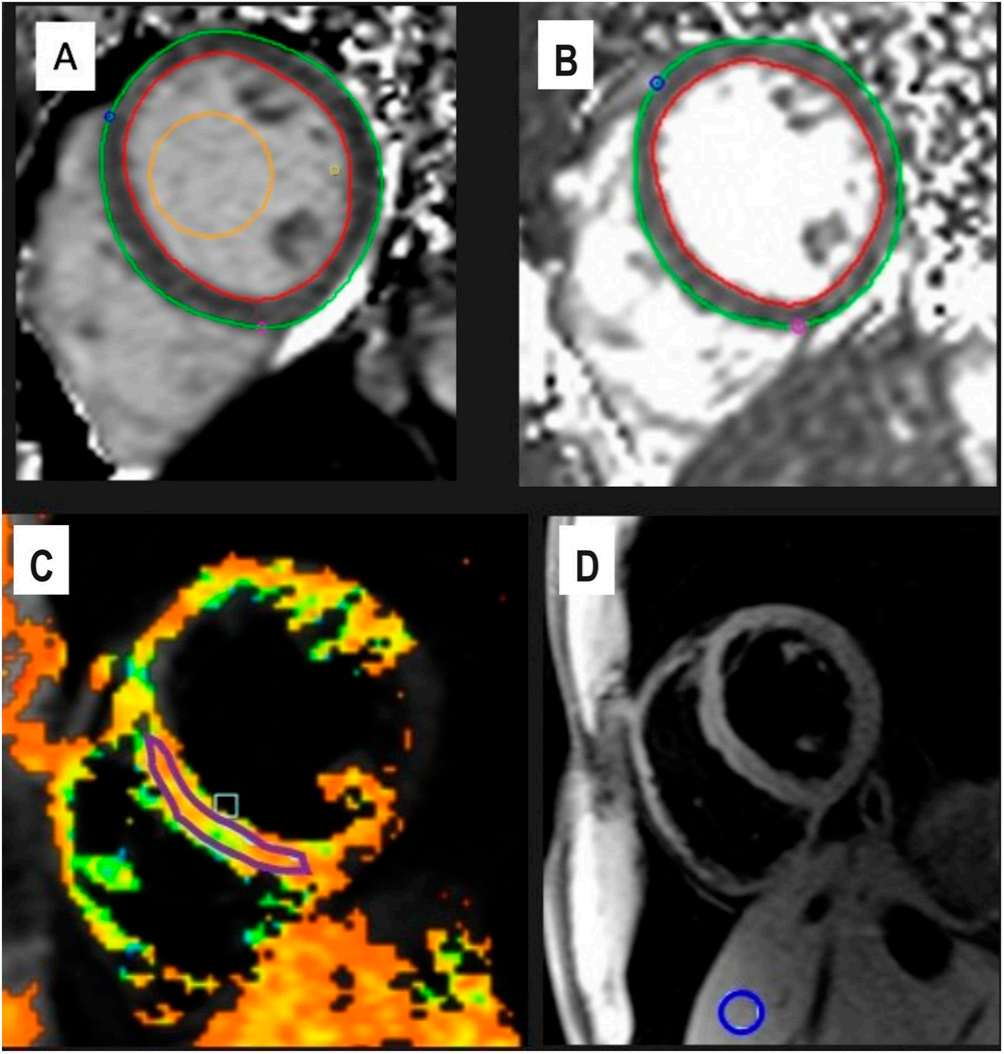
Analysis of myocardial and hepatic tissue characteristics. (a) For T1 analysis, the left ventricular (LV) epicardium (green) and endocardium (red) were automatically delineated at three short-axis levels (basal, mid-ventricular, and apical); the mid-ventricular slice is shown here. A region of interest (ROI) was placed in the LV blood pool (yellow). (b) For T2 analysis, the epicardial (green) and endocardial (red) borders were delineated identically. (c)–(d) For T2* analysis, an ROI was placed in the interventricular septum on the mid-ventricular short-axis image (purple), and a separate ROI was drawn in the liver (blue), carefully avoiding vascular structures.

Global and segmental relaxation time constants were calculated for each location in the tissue individually using Circle. The T1 (Fig. 1(a)) and T2 (Fig. 1(b)) maps were segmented using the 16-segment American Heart Association model.^36^ Regions of interest (ROI) were automatically delineated using the artificial intelligence-based contouring by Circle software by approximating the epi- and endocardial borders in the left ventricle (papillary muscles excluded). Segmental T1 and T2 values were obtained by averaging all voxels between the endocardial and epicardial borders. This approach reflects routine clinical practice in our hospital. All ROIs were verified and manually corrected when needed by an experienced imaging specialist (experience over 5 years). The T2* relaxation time constants were calculated from the mid-ventricular, apical, and basal septum by manually delineating the ROIs (Fig. 1(c)). Additionally, a reference T2* ROI was manually drawn in the liver tissue of lobus IV, avoiding veins within the same T2* map (Fig. 1(d)). Studies have shown that hepatic T2* variations are low, and the single ROI approach can be used clinically.^37^ Normal values for T1, T2, and T2* were established by calculating the mean value ± two standard deviations (2SD) for each segment for each technique, as recommended by guidelines.^35^

The participants were grouped by age and sex. Age groups were categorised as 18–30 years (*n* = 12, 50% male), 31–41 years (*n* = 14, 50% male), and 42–60 years (*n* = 14, 50% male). Statistical comparisons were made to assess differences in the T1, T2, and T2* relaxation time constants across these demographic groups and to derive age- and sex-specific normal values for the sequences. All statistical analyses were done using SPSS (version 29.0.1.0). Interobserver variability was assessed as the intraclass correlation coefficient (ICC) and coefficient of variation (CV%) in a subset of 10 randomly selected participants. Measurements were performed by two independent observers: one an experienced imaging specialist physician with over 5 years of CMR analysis experience, and the other a medical student with only a brief introductory training period in the use of Circle cvi42. Both observers independently repeated all measurements while blinded to each other’s results.

## Results

**Fig 2.**
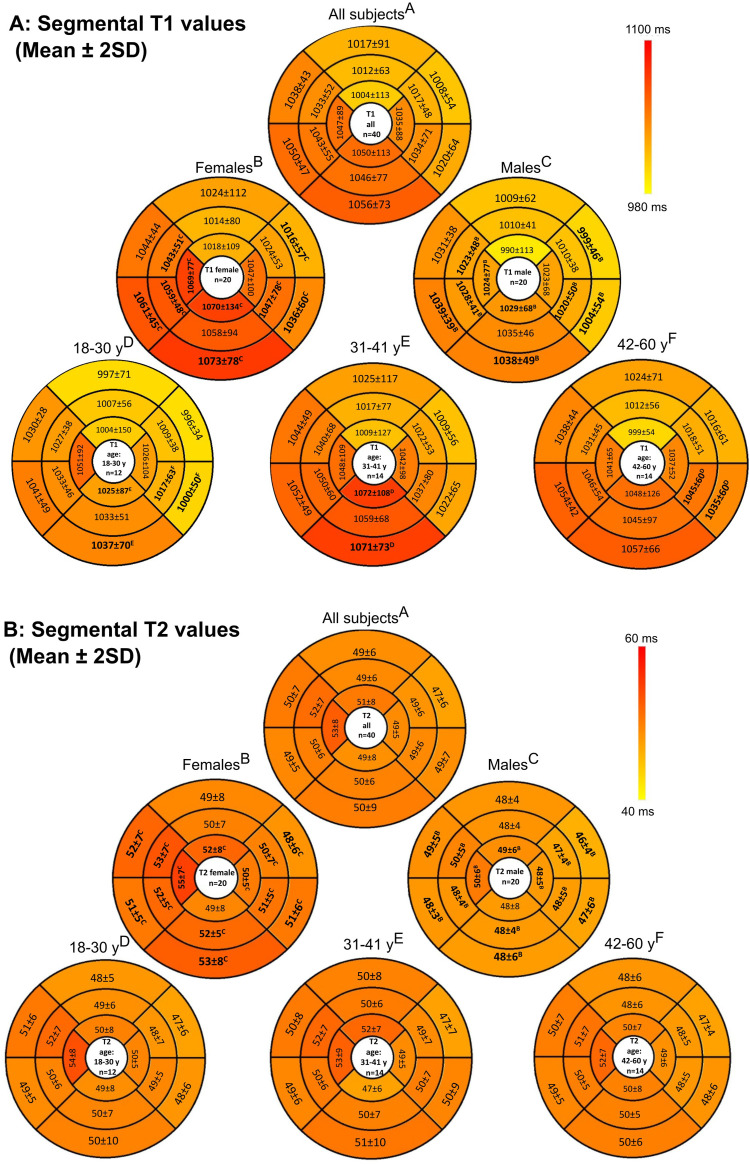
Bull’s-eye plots showing (a) T1 and (b) T2 values across 16 segments based on the American Heart Association classification. Bolded values indicate statistically significant differences (*p* < .05) between men and women or across age groups. The specific superscript letters (a)-(f) correspond to the comparison groups used to determine statistical significance. *N* = 40.

**Fig 3.**
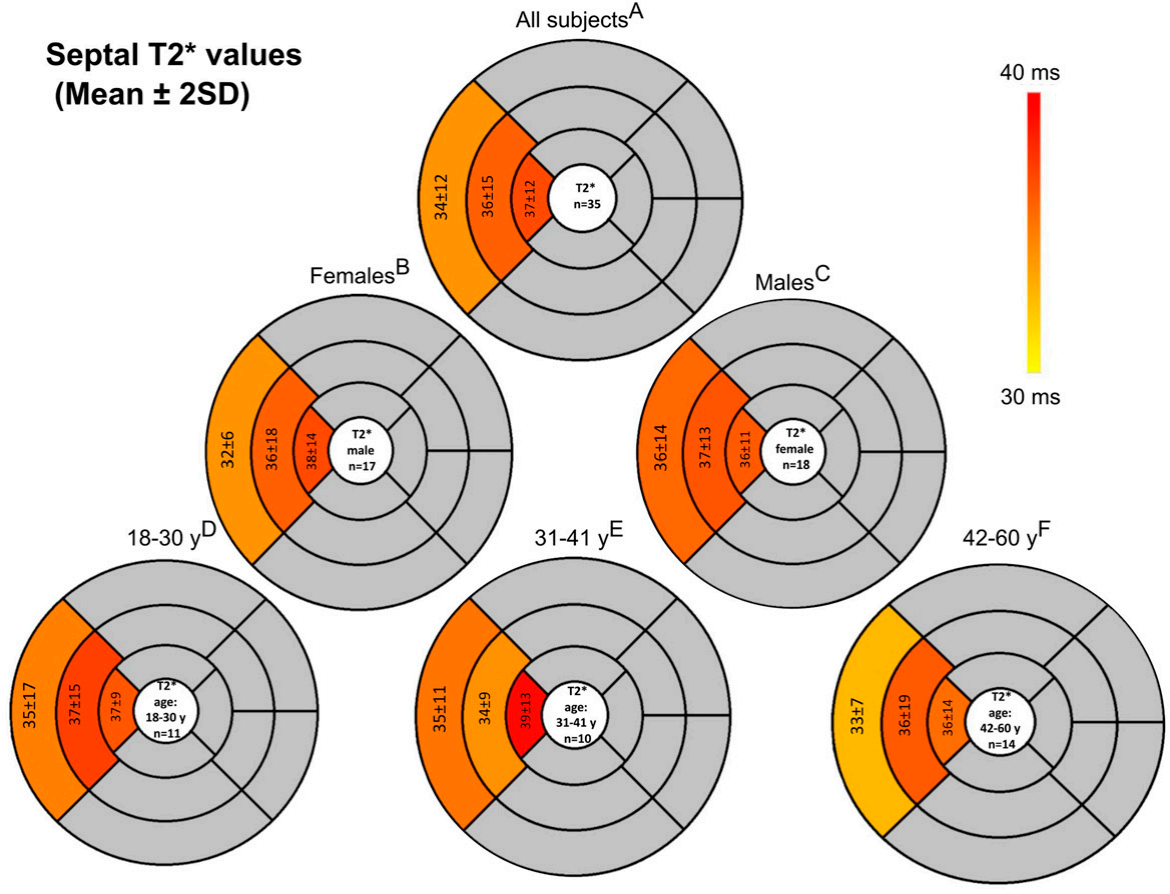
T2* results in bulls-eye plots. T2* values are measured only from the septum. *N* = 35.

The overall population demographics can be seen in Table 2. Women had higher T1 and T2 relaxation times compared to men, as shown in Table 3. Differences were observed in the inferior, septal, and lateral walls but not in the anterior wall (Fig. 2). There was no difference in the T2* relaxation time constants between the sexes in either the myocardium (Fig. 3) or the liver.

**Table 2.** Characteristics.

	All	Females	Males	*p*-value	Age 18-30 y (a)	Age 31-41 y (b)	Age 42-60 y (c)	*p*-value
	*N* = 40	*N* = 20	*N* = 20		*N* = 12	*N* = 14	*N* = 14	
	Med. (range)	Med. (range)	Med. (range)		Med. (range)	Med. (range)	Med. (range)	
Age (years)	33 (23-60)	34 (23-54)	33 (23-60)	Ns.	28 (23-29)	33 (31-41)	49 (42-60)	(a-b, a-c, b-c)***
Weight (kg)	77.0 (57.5-130.0)	74.5 (57.5-96.5)	83.7 (59.0-130.0)	**	75.8 (57.5-130.0)	76.5 (59.0-110.0)	81.1 (64.5-97.9)	Ns.
Height (cm)	175 (156-188)	171 (156-177)	180 (173-188)	**	175 (156-180)	175 (163-186)	173 (160-188)	Ns.
BMI (kg/m^2^)	26 (18-40)	26 (21-35)	26 (18-40)	Ns.	25 (21-40)	24 (18-32)	27 (21-35)	(b-c)*
WHR (*n* = 39)	0.8 (0.7-1.2)	0.8 (0.7-0.9)	0.9 (0.8-1.2)	***	0.8 (0.7-1.2)	0.8 (0.8-1.0)	0.9 (0.8-1.1)	Ns.
SystolicBP (mmHg)	125 (105-150)	120 (105-150)	135 (105-150)	***	125 (107-134)	120 (105-149)	138 (109-150)	(a-c, b-c)**
DiastolicBP (mmHg)	81 (66-104)	80 (66-104)	84 (72-100)	Ns.	80 (69-100)	77 (66-96)	87 (73-104)	(a-c)*, (b-c)**
Haemoglobin (g/l)	146 (124-172)	137 (124-151)	155 (136-172)	***	150 (124-172)	139 (124-168)	147 (127-164)	Ns.
LDL (mmol/l)	2.6 (1.3-4.2)	2.4 (1.3-3.5)	2.6 (2.0-4.2)	**	2.4 (1.9-3.2)	2.6 (1.3-2.6)	2.7 (2.0-4.2)	(a-c)*
CKMB (ug/l)^†^	1.7 (0.8-6.0)	1.5 (0.8-4.7)	2.1 (1.0-6.0)	*	1.6 (1.0-4.7)	1.6 (0.8-4.8)	2.1 (1.1-6.0)	Ns.
Hba1c (mmol/l)	33 (29-43)	33 (30-37)	34 (29-43)	*	32 (29-36)	34 (32-38)	35 (31-43)	(a-b, a-c)**
proBNP (ng/l)^†^	27 (<10-141)	34 (<10-71)	18 (<10-141)	*	26 (<10-59)	31 (<10-136)	26 (<10-141)	Ns.
hs-CRP (mg/l)^†^	0.6 (<0.3-7.0)	0.7 (<0.3-7.0)	0.6 (<0.3-2.3)	Ns.	0.7 (<0.3-5.1)	0.4 (<0.3-7.0)	0.8 (<0.3-6.7)	Ns.
PR time (ms)	158 (116-200)	142 (116-195)	172 (135-200)	**	144 (117-195)	152 (116-200)	160 (122-197)	Ns.
QRS (ms)	97 (80-115)	94 (80-106)	100 (87-115)	**	96 (81-105)	95 (80-109)	97 (82-115)	Ns.
QTc (ms)	411 (364-451)	415 (285-451)	405 (364-442)	*	402 (374-437)	399 (364-429)	414 (380-451)	(b-c)*
LVEF (%)	61 (47-68)	61 (54-68)	59 (47-67)	Ns.	61 (53-68)	61 (54-66)	61 (47-67)	Ns.
LVEDV_index_ (ml/m^2^)	89 (66-120)	86 (66-118)	91 (72-120)	Ns.	88 (80-115)	88 (66-120)	85 (69-105)	Ns.
LVESV_index_ (ml/m^2^)	36 (23-69)	35 (23-49)	38 (24-69)	*	38 (29-56)	38 (23-69)	35 (24-45)	Ns.

y = years, BMI = Body Mass Index, WHR = Waist-to-Hip Ratio, Systolic BP = Systolic Blood Pressure, Diastolic BP = Diastolic Blood Pressure, LDL = Low-Density Lipoprotein, CKMB = Creatine Kinase-MB, HbA1c = Haemoglobin A1c, proBNP = Pro B-type Natriuretic Peptide, hs-CRP = High-Sensitivity C-Reactive Protein, PR time = Time from the start of the P wave to the start of the QRS complex, QTc = Heart rate Corrected QT Interval, LVEF = Left Ventricular Ejection Fraction, LVEDV_index_ = Left Ventricular End-Diastolic Volume Indexed to body surface area, LVESV_index_ = Left Ventricular End-Systolic indexed to body surface area. Statistical differences between age groups (a-c) and presented: *p < .05, **p < .01, ***p < .001. Ns. = non-significant. † = non-normally distributed.

**Table 3.** T1, T2, T2* results in all patients, males and females.

	Total (*n* = 40)	Males (*n* = 20)	Females (*n* = 20)	*p*-value
	Mean ± SD	Mean ± SD	Mean ± SD	
T1 value (ms)				
Base	1031.2 ± 36.4	1020.1 ± 29.0	1042.4 ± 19.8	***
Mid	1030.9 ± 33.0	1020.9 ± 11.8	1041.0 ± 19.0	**
Apex	1033.8 ± 53.3	1016.8 ± 21.9	1050.8 ± 28.4	**
Global	1029.8 ± 25.8	1019.9 ± 17.3	1039.7 ± 29.2	**
T2 value (ms)				
Base	49.1 ± 2.3	47.7 ± 1.6	50.4 ± 2.1	***
Middle	50.1 ± 3.0	48.5 ± 2.2	51.7 ± 2.9	***
Apex	50.2 ± 2.8	48.7 ± 2.1	51.7 ± 2.6	***
Global	49.6 ± 3.5	48.2 ± 2.6	51.1 ± 3.7	***
T2* myocardial (ms)				
Base (*n* = 34)	34.2 ± 5.8	32.3 ± 2.9	35.8 ± 7.2	Ns.
Mid (*n* = 35)	36.1 ± 7.8	35.6 ± 9.0	36.6 ± 6.5	Ns.
Apex (*n* = 35)	37.0 ± 6.2	37.6 ± 6.9	36.4 ± 5.6	Ns.
Global (*n* = 35)	35.8 ± 6.7	35.2 ± 7.1	36.3 ± 6.4	Ns.
T2* liver (ms) (*n* = 35)	28.7 ± 5.4	27.6 ± 4.1	29.6 ± 6.3	Ns.

*p*-values are calculated between males and females. Statistical difference in studied between sex are presented as **p < .01, ***p < .001, Ns. = non-significant. SD = standard deviation.

The participants were divided into three age groups (18–30 years, 31–41 years, and 42–60 years). The global myocardial T1, T2, and T2* relaxation time constants did not differ significantly between the age groups (*p* = .45–.70). However, a significant difference was found in the basal T1 relaxation times, with lower values observed in the 18–30 age group compared to the two older groups (Table 4). When age groups were analysed by sex, no significant differences in the global T1, T2, or T2* times were noted, although a few segments showed significant differences (*p* < .05, Fig. 2).

**Table 4.** T1, T2, T2* results in different age groups.

	18-30 y (a) (*n* = 12)	31-41 y (b) (*n* = 14)	42-60 y (c) (*n* = 14)	*p*-value
	Mean ± SD	Mean ± SD	Mean ± SD	
T1 value (ms)				
Base	1016.7 ± 32.2	1037.4 ± 40.8	1037.4 ± 31.9	(a-b,a-c)**
Mid	1021.0 ± 26.3	1037.4 ± 36.2	1033.0 ± 33.5	Ns.
Apex	1026.5 ± 56.2	1042.6 ± 58.6	1031.2 ± 44.3	Ns
Global	1021.8 ± 19.8	1032.8 ± 34.8	1033.6 ± 19.1	Ns
T2 value (ms)				
Base	48.8 ± 1.4	49.5 ± 3.0	48.9 ± 2.3	Ns.
Middle	50.0 ± 2.8	50.8 ± 3.3	49.6 ± 3.0	Ns.
Apex	50.5 ± 3.1	50.3 ± 2.4	49.8 ± 2.9	Ns.
Global	49.6 ± 3.6	50.0 ± 3.8	49.3 ± 3.2	Ns.
T2* myocardial (ms)				
Base (*n* = 34)	35.0 ± 8.4	35.4 ± 5.4	32.8 ± 3.7	Ns.
Middle (*n* = 35)	37.4 ± 7.6	34.3 ± 4.7	36.5 ± 9.6	Ns.
Apex (*n* = 35)	36.5 ± 4.5	39.4 ± 6.3	35.7 ± 7.2	Ns.
Global (*n* = 35)	36.3 ± 6.8	36.3 ± 5.7	35.0 ± 7.3	Ns
T2* liver (ms) (*n* = 35)	30.9 ± 5.7	29.4 ± 4.4	26.1 ± 5.0	(a-c)**

Statistical differences between age groups are presented as **p < .01, ***p < .001, Ns. = non-significant. SD = standard deviation.

Normal reference values, defined as mean ± 2SDs, are presented in Table 5. The normal T1 range was 981–1098 ms for females and 985–1054 ms for males. The upper limit for normal T2 values was 58 ms for females and 53 ms for males. The lower limit for normal T2* values in the septal region for the entire population was 21 ms.

**Table 5.** Normal values for T1, and T2, values are represented as global, septal, inferior, lateral, anterior, and apical segments.^36^

	All (ms)	Males (ms)	Females (ms)
T1 Global	978–1081	985–1054	981–1098
Septal^2,3,8,9^	990**–**1091	988**–**1073	1003**–**1101
Inferior^4,10^	976**–**1126	990**–**1084	979**–**1152
Lateral^5,6,11,12^	957**–**1082	959**–**1057	965**–**1097
Anterior^1,7^	936**–**1092	957**–**1061	922**–**1116
Apical^13–16^	927**–**1140	929**–**1104	937**–**1164
T2 Global	43–57	43–53	44–58
Septal^2,3,8,9^	44**–**57	44**–**53	46**–**58
Inferior^4,10^	43**–**57	43**–**53	45**–**59
Lateral^5,6,11,12^	42**–**55	42**–**52	44**–**56
Anterior^1,7^	43**–**55	44**–**52	42**–**57
Apical^13–16^	42**–**58	42**–**55	44**–**60
T2*mid septal myocardial	21-52	18-54	24-50
T2* liver	18-40	19-36	17-42

Septal, inferior, lateral, anterior, and apical segments are defined from the AHA 16-segment model.^36^ Two-sided normal range is calculated for T1 and T2 values as mean +/− 2 standard deviations.

Interobserver variability was assessed in 10 participants and was overall low, demonstrating good reproducibility and agreement between measurements performed by an experienced and a novice user of the Circle CVI software (Table 6).

**Table 6.** Interobserver variability results.

	Cronbach’s alpha	Interobserver reproducibility (ICC)	Coefficient of variation (CV%)
T1 Global	0.86	0.73	1.3
T1 Basal	0.99	0.98	0.3
T1 Middle	0.99	0.97	0.3
T1 Apex	0.99	0.96	0.7
T2 Basal	0.97	0.94	0.8
T2 Middle	0.99	0.98	0.5
T2 Apex	1.00	0.99	0.5
T2* Basal (*n* = 9)	0.90	0.77	5.4
T2* Mid	0.98	0.96	4.6
T2* Apex	0.60	0.41	13.0
T2* Liver	0.89	0.81	7.9

## Discussion

Our findings indicate that women have higher myocardial T1 and T2 values compared to men, with differences evident in the inferior, septal, and lateral walls but not in the anterior wall. However, no sex differences were observed in the T2* relaxation time constants, either in the myocardium or in the liver. Furthermore, age did not affect the global myocardial T1, T2, or T2* relaxation times, illustrating that these values remained stable across the studied age groups.

Typically, values from healthy subjects in CMR tissue characteristics are reported as combined results for both men and women. Our findings confirmed that women have higher T1 and T2 relaxation times than men, consistent with previous literature suggesting biological rather than technical origins for these differences. In our study, the T1 range (mean ± 2 standard deviations) was 981–1098 ms for females and 985–1054 ms for males. For the T2 relaxation times, we established an upper limit of 58 ms for females and 53 ms for males. Potential explanations for why women had higher T1 and T2 might include hormonal influences, such as the effects of oestrogen on myocardial tissue composition, reduced intramyocardial blood haematocrit, differences in water content, fat distribution, extracellular volume fraction, or subtle structural variations in the myocardium.^38^ Previous studies have shown that native T1 and extracellular volume are influenced by sex.^20^ In addition, cardiac chamber size and heart rate have been shown to further modulate these values,^20^ highlighting that myocardial relaxation parameters are shaped not only by intrinsic tissue characteristics but also by physiological loading conditions.

Our study design closely resembles that of Cadour et al., who also evaluated myocardial T1, T2, and T2* values in healthy volunteers at 1.5 T using comparable mapping sequences.^38^ Consistent with our findings, they reported higher T1 and T2 values in women. However, unlike our cohort, Cadour et al. observed a clear age-related decline in both parameters and noted that sex-related differences narrowed with increasing age. Several factors may explain these divergent results. Their study included a larger and more evenly distributed age sample, providing greater power to detect modest age-related changes. In contrast, our upper age group likely included both pre- and post-menopausal women, introducing hormonal heterogeneity that we could not stratify due to our relatively small sample size.

Other previous studies have also evaluated healthy subjects with CMR tissue-characterisation. For example, Granitz et al. reported slightly lower T1 values (934-1058 ms) than us,^39^ Abdullah et al. documented T1values ranging from 946 to 1078 ms.^40^ Granitz et al. found a higher combined upper T2 limit of 62 ms,^39^ whereas Meloni et al. reported an upper T2 value of 58 ms.^41^ We determined that the combined normal lower limit for T2* in the combined population of males and females was 21 ms, consistent with prior results indicating that values above 20 ms are considered normal.^42^ The T2* values are generally reported as not being sex-related, and our results align with that statement.^35^ Additionally, our study included liver T2* measurements, which fell within the range of previously reported values.^34^ No difference was observed between the sexes.

The influence of age on the T1, T2, and T2* relaxation times remains inconsistent in the literature. Some studies suggest a slight increase with age,^40^ while others report no differences,^30^ similar to our findings. To ensure the validity of our results, we thoroughly screened participants to rule out underlying cardiac diseases, as undetected pathologies could potentially skew findings, particularly in older populations where such conditions are more prevalent. In our study population, we observed no other consistent age-related trends apart from an increase in HbA1c levels rising with age (Table 2). It is possible that the older patients in our study were exceptionally healthy, as cardiac diseases had been ruled out. This may have minimised the typical physiological changes associated with ageing. As a result, age-related differences in myocardial relaxation times may not have been apparent in this population. This could either suggest a selection bias towards healthier individuals or indicate that the physiological impact of ageing on these parameters is less pronounced in those with optimal cardiovascular health.

We also evaluated regional differences in values and observed clear regional variation in T1 and T2 values across the American Heart Association (AHA) 16-segment model. Septal and inferior segments tended to show slightly higher T1 and T2 ranges compared with lateral and anterior walls, while the apical segments exhibited the broadest ranges, particularly in women. These findings were consistent with the basal–mid–apical pattern seen in the three short-axis levels, where apical T1 and T2 values were generally higher and more variable than those in the mid-ventricular or basal slices. This pattern can be explained by a combination of biological and technical factors. Anatomically, regional differences in myocardial fibre orientation, wall thickness, strain patterns, perfusion, and extracellular volume fraction may contribute to modest physiological variation in relaxation times.^35^ Technically, the apex is particularly susceptible to partial-volume effects due to its thinner wall and proximity to the blood pool, and it experiences greater curvature and more complex systolic motion, all of which may elevate measured T1 and T2 values and widen their variability.^43^ Mid-ventricular and basal segments, with their thicker myocardium and more stable imaging geometry, therefore, yield more homogeneous values. The slightly higher T2 values in apical and mid-ventricular regions may further reflect both these technical influences and intrinsic differences in strain and tissue composition across the ventricle. Collectively, these findings highlight that segmental and level-dependent differences in T1 and T2 mapping are expected in healthy myocardium and should be considered when interpreting region-specific measurements. In contrast, T2* values showed no systematic gradient from base to apex.

The biggest limitation of our study is the relatively small sample size (40 volunteers), constraining the statistical power. Additionally, we focused on adults aged 18–60 years, excluding both younger and older individuals and potentially diminishing the age effect by omitting older cohorts. Consequently, the age group represents a mixture of male and female values, which may mask true age-related differences. Thus, the absence of an observed age effect should be interpreted with caution. Also, an important limitation of this study is that the oldest age group (42–60 years) likely included both pre- and post-menopausal women, introducing heterogeneity in hormonal status. Post-menopausal decline in oestrogen is known to affect myocardial structure and extracellular matrix biology.^44^ These hormonal changes could potentially influence myocardial water content and extracellular volume, thereby partially contributing to differences in relaxation times. This is supported by recent findings from Cadour et al.,^38^ who reported age-related decreases in myocardial tissue-characterisation values among women, consistent with a potential menopausal effect. However, given the limited sample size in our cohort, we were unable to meaningfully stratify women by menopausal status, and this information was not collected or assessed through laboratory measures. This remains an important consideration for future studies. One of the strengths of this study was the thorough exclusion of cardiac diseases and conditions that could have led to a bias in the results. We also assessed interobserver variability, which was generally low, enhancing the reliability of our measurements. Future research with larger, more diverse populations, including older adults, and standardised imaging protocols could be of value to develop more comprehensive reference ranges for the T1, T2, and T2* relaxation times in cardiac MRI.

In conclusion, these findings highlight the need for sex-specific interpretation of T1 and T2 so that physiologically higher values in women are not misclassified as abnormal.
